# Adoption of environmental management accounting in Vietnamese enterprises: An empirical analysis of influencing determinants

**DOI:** 10.1371/journal.pone.0304902

**Published:** 2024-07-25

**Authors:** Thi Mai Anh Nguyen

**Affiliations:** School of Accounting and Auditing, The National Economics University (NEU), Hanoi, Vietnam; Guangxi Normal University, CHINA

## Abstract

Along with the process of rapid economic development and integration, Vietnam is facing serious environmental problems, largely caused by manufacturing enterprises. Environmental management accounting (EMA) is a strong management tool to improve an organization’s financial and environmental performance, hence supporting businesses for sustainability. This study focused on examining the factors that influence the firms’ intention to adopt EMA in Vietnam. The study used the theory of planned behavior and the technology acceptance model to develop an empirical model and hypotheses. Research data was collected through a survey of 330 businesses in 4 provinces in the North region. We analyzed the data using descriptive statistics, Anova test, Cronbach’ s Alpha analysis, Exploratory factor analysis, and regression. The results showed that there are 6 significant factors driving EMA applying intention including environmental attitude, perceived benefits of EMA, subjective norms, coercion and social pressure, perceived control and barrier, and policy mix condition, in which coercion and social pressure was the strongest influencing factor. From there, the study makes some implication for government and business sectors aimed at encouraging the implementation of EMA in Vietnam.

## 1. Introduction

In the process of economic development, serious consequences due to resource degradation and environmental pollution have caused many countries to pay special attention to environmental protection towards sustainable development [1–3]. At the enterprise level, to improve the quality and effectiveness of environmental management, accounting plays an important role. In that context, environmental management accounting (EMA) was born as a necessity to meet the requirements for environmental information in business activities towards business efficiency [4, 5]. EMA is a component of corporate management accounting that involves collecting, processing, analyzing and providing environmental information for internal and external stakeholders to use for decision making [6, 7]. Unlike traditional accounting, EMA not only focuses on financial aspects but also focuses on evaluating and managing the impacts of business activities on the environment. EMA is becoming increasingly important in the context that businesses and society want to achieve efficiency and sustainability [8, 9].

Currently, companies are increasingly aware of the environmental impact of their operations, products and services. Environmental risks cannot be ignored; they play an important role in running a successful company as well as in product design, marketing and sound financial management [10, 11]. Poor environmental behavior can have a real negative impact on a company and its finances. Penalties include fines, increased environmental tax liability, loss of property value, damage to brand value, loss of revenue, consumer boycotts, lack of financing options, and loss of insurance, liability, litigation and damage to the company’s image. Today, almost every aspect of the economy is affected by environmental issues, including management accounting [11, 12]. From an accounting perspective, the first pressures are felt in external reporting, including the disclosure of environmental aspects in financial statements and/or the preparation of separate environmental reports. Much has been written about the nature and quality of these accounts. However, environmental problems cannot be solved by external reporting alone. Before environmental problems can be reported, they must be managed, and this requires changes to the accounting system [9].

Vietnam is in the group of countries that are developing and integrating very strongly [13]. According to the World Bank (2022), Vietnam’s average economic growth rate in the period 2000–2020 is 7.2%, among the highest in the Asia Pacific region [14]. However, environmental pollution has always been considered a serious problem for many years [13, 15]. Vietnam is currently ranked 160 countries in the world in terms of environmental quality according to the Environmental Performance Index (2019). Reality has proven that, in a modern economy, failure to collect data related to environmental impacts on business activities will cause certain consequences in the use of environmental impacts information for decision-making at all levels of enterprise management [15, 16]. In this situation, EMA is a method that can meet the goal and relieve the above environmental and economic pressures in Vietnam. This tool synthesizes many different technical platforms such as full cost accounting, life cycle costing, environmental budgeting, and evaluation of environmental capital investments. On the one hand, this helps managers allocate environmental costs that traditional accounting often overlooks and treats as a general production cost. On the other hand, it provides both monetary and non-monetary data on factors related to the environment for managers to orient and evaluate the nature of opportunities and opportunities to improve environmental and economic performance [14–16].

Vietnam is also restructuring its economy and promoting rapid and sustainable growth. Like in many other nations, environmental information is increasingly concerned by stakeholders and creates pressure on businesses to implement EMA [17]. In fact, identifying and recording environmental costs has been done by businesses for a long time but is not complete and has not become a formal accounting system. Along with the development of the economy, political institutions and increasing needs of all parties, the application of EMA has become more popular among Vietnamese businesses, but mainly takes place in large and medium manufacturing enterprises and the content of the new EMA focus on the aspect of cost management [18]. However, it is a good sign that there is a certain similarity in EMA practice in Vietnam with countries around the world [14–18].

In literature, many authors have undertaken EMA related studies and proposed policy implications for countries in enhancing management accounting institutions to increase transparency, efficiency and sustainability of businesses [4, 9, 19, 20]. However, the analysis of behaviors and factors affecting the intention to apply EMA is overlooked in accounting policy research, thereby leading to a lack of adequate information for the process policy planning and management tools [21]. In particular, a review of EMA literature [9, 20, 21] pointed out that research on EMA adoption behavior is lacking in developing countries like Vietnam and hence lack of information to make management decisions.

This study, therefore, aims for bridge this gap by identifying and analyzing the impact of factors involving the intention to apply EMA in businesses in Vietnam. Currently, in the country, there is still a lot of controversy surrounding the benefits and impacts of EMA and there are very few empirical studies on EMA at the enterprise level [18]. Such a study hence can contribute to promoting the current development of EMA in Vietnam. The study also provides empirical evidence on the factors influencing EMA adoption in countries with rapid economic transition and allows comparing the similarities and differences with the flow of this research direction around the world.

## 2. Theoretical framework and hypothesis development

### 2.1 Theoretical framework

The term EMA first appeared at the Environmental Summit in Stockholm (1972). According to the International Federation of Accountants (IFAC), ‘EMA is the process of managing economic efficiency and environmental efficiency through the development and application of accounting systems appropriate to environmental issues. EMA is a part of corporate management accounting, aiming to collect, process, analyze and use monetary and physical information related to the impact of businesses on the environment, thereby improving operations of the business in both financial and environmental aspects [22]. The United Nations Sustainable Development Agency (UNDSD) also defines ‘EMA as a specific aspect of management accounting. The general purpose of environmental accounting is to provide information for decision making including material calculations such as raw materials, energy consumption, material flows and the amount of material removed or emissions, costs, and revenues related to activities that have the potential to affect and potentially impact the environment’ [23].

EMA is applied in businesses to achieve potential benefits including (i) fully identify, accurately determine and properly allocate environmental costs to help price products and determine results doing business correctly, thereby making reasonable decisions on product strategy as well as investment in equipment and technology; (ii) control operating costs and improve the environment thanks to controlling waste associated with the source; (iii) improve relationships with creditors, banks, shareholders, and customers by meeting international environmental standards, creating commercial advantages, and enhancing reputation in the community through image development "green"; (iv) improving the existing accounting system by organizing the accounting information system more scientifically and connecting the information flow of activities from all departments of the enterprise [9, 19, 20].

According to Xiaomei (2014), if one could identify variables that impact the adoption of a management tool such as EMA and if one could predict adoption with a certainty, it could dramatically increase the reasonable EMA adoption by decisive interventions [9]. So far, researchers often use technology-organization-environment framework (TOE) as theoretical framework to explain management tool adoption in organizations and describe how the process of adopting innovations are influenced by socio, economic and environmental contexts [9, 19, 24]. The framework focuses on higher level attributes instead of detailed behaviors of individuals in the organization. As to Schaltegger and Petersen (2013), to understand tools/technology adoption at individual level, behavioral models such as the theory of reasoned action, the theory of planned behavior, and the technology acceptance model should be applied [24]. Bebbington et al. (2017) demonstrated a rough equivalence of behavioral models and TOE framework when individual perception has been taken into account [2020]. This study coordinates TOE, TRA, TPB and TAM to build a research model. Essentially, the model attempted to treat the dependent variables of EMA adopt intention of firms as a function of contextual constructs and develop a predictable understanding of the relationships for impactful business implications and policy.

#### Theory of reasoned action (TRA)

TRA is a prediction model of behavioral intention, considering intention as a continuation between attitude and behavior, built by Fishbein and Ajzen (1989). This theory shows that behavioral intention is the most important factor to predict consumer behavior and is influenced by two factors—attitude and subjective norms [25]. Behavioral intention considers the subject’s subjective likelihood of performing a behavior and can be viewed as a special case of belief. Attitude is the attitude toward an action or behavior that represents an individual’s positive or negative perception and is measured by the combination of the carrying capacity of beliefs and evaluations of this belief. If the outcome is beneficial, they may intend to engage in the behavior [26, 27]. Subjective norms are understood as an individual’s perception of whether the individual should or should not perform a behavior. Subjective norms can be measured through the relevant people’s defined normative beliefs about the expected performance of the behavior and the individual’s motivation to perform in accordance with that expectation. Thus, the TRA theory predicts that behavioral intentions depend on the individual’s behavior and subjective standards of the surrounding environment. The model assumes that people make rational decisions based on available information to perform or not perform a behavior. TRA provides a simple tool to identify individual behavior [25–27].

#### Theory of planned behavior (TPB)

Ajzen (1991) improved TRA model by adding a new variable "Behavioral control" in the impact equation [26]. It represents the resources needed to perform any job, the resources, skills, opportunities and perceptions of each individual that influence behavior and achieve results. This variable is affected by two variables: control beliefs and perceived ease. Control beliefs are understood as individuals feeling confident to perform behavior [27]. Three important determinants in this theory are the attitudinal factor which is the individual’s attitude towards the behavior in terms of the positive or negative of performing the behavior, in terms of perceived intention of social pressure of the person. The person, because it deals with the perception of pressure or normative compulsion, is called the subjective norm, and is ultimately the determining factor in self-awareness or ability to perform behavior, known as cognitive behavioral control [26, 28]. The theory suggests the importance of attitudes toward behavior, subjective norms, and perceived behavioral control leading to the formation of a behavioral intention [27, 28].

#### Technology acceptance model (TAM)

this model was built by Davis in 1989, based on the development of TRA and TPB theory [29]. The model explains the factors influencing technology acceptance and technology user behavior on the basis of TRA theory. In the TAM model, the factors identified include perceived ease of use, perceived usefulness, attitudes that influence users’ intention and behavior in accepting information technology [29]. Research results by Bhattacharyya (2011) also showed that the TAM model explains 40% of the likelihood of success of the intention to apply new technology and systems. In particular, perceived usefulness is the ability of individuals to believe that using a system or technology will improve their performance. Perceived ease of use is the individual’s ability to believe that using a technology or system will be effortless [29, 30].

### 2.2 Hypothesis development

Researches around the world on EMA application determinants were mostly carried out based on the above theories, in which empirical models were built based on specific situations. This study’s model hence was also designed based on theories, literature and Vietnamese context to identify impacted factors of EMA adopting intention. The next section introduces key anticipatory factors in the empirical models based on an overview of studies on planned behavior, business and environment.

#### Environmental attitude (EA)

According to Tarkiainen et al. (2015), environmental attitude is "the positive or negative feelings of individuals towards issues related to the environment that come from their awareness" [31]. Al (2013) suggested that “Environmental attitude is the concern for environmental issues that stems from each individual’s own perspective and the degree to which that individual perceives himself to be part of the environment’ [32]. TPB theory states that attitude is an important component that directly affects the intention to perform a certain behavior [25]. This assumption has been proven in many studies on corporate behavior and the environment [33–35]. Basically, when a business’ attitude about environment increases, the likelihood to adopt environmentally friendly behaviors will also increase. Maama and Appiah (2019) found that businesses’ environmental attitudes closely relate to their awareness and evaluation of environmental issues that can affect business performance or corporate sustainability [36]. In addition, Modell (2014) indicated that attitudes not only include subjective perceptions and feelings about the good or bad state of environmental issues but also involve the compatibility of values that businesses can bring with their friendly behaviors [37].

*Hypothesis H1*: *Environmental attitude has a positive impact on the intention to adopt EMA*.

#### Perceived benefit (PB)

Perceived benefit is defined as a user’s perception of the potential that a tool/technology has to bring benefits to the organization when applied [38]. Many studies have proven that perceived benefits have a positive impact on intention to use technology such as Schaltegger et al. (2012), Wild and Van Staden (2012), Vejzagi et al. (2018). More specifically, Modell (2014) demonstrated that perceived benefits have a positive impact on the intention to apply EMA in businesses in Australia. Andon et al. (2015) also believed that awareness of the benefits of management solutions might affect the choice of policies and strategies in business activities. In business management, when managers have clear benefit awareness and high consensus, demonstrated by strategic commitments and specific actions to improve business performance; it might create motivation for enterprises to apply EMA [38–41].

*Hypothesis H2*: *Perceived benefit has a positive impact on the intention to adopt EMA*

#### Subjective norm (SN)

Subjective norm is a person’s perception of social pressure to perform or not perform a behavior. When a person performs a specific behavior, they might perceive the praise, criticism, and judgment of society or relatives for that behavior [42]. It is these pressures that hinder or motivate them to perform the behavior. Ajzen (1991) stated that subjective norms are social pressures that encourage a person to engage in a particular behavior [26]. Subjective norm is a common behavioral norm followed by a social group. That is, subjective norms are a measure of social group pressure that an individual must take into account before making behavioral decisions [43]. Cordeiro and Sarkis (1997) believed that it is necessary to redesign traditional management accounting systems to meet the increasing demand for environmental information. If an organization experiences high levels of business environment volatility, it may innovate the accounting system to accommodate the need for additional information to cope with unforeseen change [44]. These innovative systems aim to provide environmental information to manage costs and minimize environmental impacts. Kwakye et al (2018) showed that subjective standards have a positive influence to implement EMA [45]. Setthasakko (2017) pointed out factors affecting the implementation of EMA at enterprises in Thailand including subjective standards, pressure from stakeholders, awareness of senior managers, accounting staff qualifications on environmental information, business characteristics and coercive pressure from state management agencies [46]. In particular, subjective norms have the strongest influence, followed by coercive pressure and perceptions of senior managers [47].

*Hypothesis H3*: *Subjective norm has a positive impact on the intention to adopt EMA*.

#### Coercion and social pressure (CSP)

Previous studies have examined the factors that drive UKORE from both external and internal perspectives [4, 9]. As for external drivers, many scholars argue that companies engage in green management to respond to external pressures from various stakeholders, such as government regulations, regional politics, community, industry standards, and customer needs [9, 18]. In addition to the desire to respond to external pressures, others see a role for internal drivers such as company characteristics, leadership values, and strategic direction [33, 37]. Previous studies combine intrinsic and extrinsic motivations to demonstrate comprehensive motivations from a complementary perspective. For example, Olaoye and Olaipekun (2018) examined the impact of external pressure and internal capabilities on a company’s proactive environmental activities [47]. Yakhou M, Dorweiler (2014) analyzed the impact of external pressure, internal motivation and the interaction between them on the implementation of environmentally friendly behavior in companies [48].

Some studies applying TPB and TOE indicated that coercive pressures have significant impacts on the application of EMA [27, 32, 49]. If the government and functional agencies strictly control environmental regulations or have policies to encourage businesses to implement environmental commitments, there would be a higher possibility of EMA adoption. Bhattacharyya (2011) opened this line of studies by showing that EMA application depends on the enterprise’s institutional environmental strategy [4]. Le (2019) demonstrated that the application of EMA is influenced by the institutional and social context related to the increase in environmental regulations [18]. Tarkiainen et al. (2015) in their research in Australia identified four factors affecting the development of EMA including interdisciplinary connections between departments in the enterprise, environmental commitments, pressures from legal regulations and markets [31]. Similarly, Maama and Appiah (2019) discovered 4 factors affecting the EMA application in Europe including environmental strategy, coercive pressure, manager awareness and external supports [36].

*Hypothesis H4*: *Coercive and social pressure has a positive impact on the intention to adopt EMA*

#### Perceived behavior control (PBC)

PBC is an individual or organization’s perception of the convenience or difficulty of performing a certain job. If they feel having enough resources and opportunities, they will be more likely to intend to perform the behavior [26, 28]. As to Ajzen (1991), perceived control factor comes from the confidence of the individual who intends to perform the behavior and the easy and favorable conditions for performing the behavior [28]. Shah and Mohamed (2011) suggested that resource limitations and self-efficacy are two constructs capable of measuring behavioral control. Resource limitations refer to the availability of resources while self-efficacy refers to a person’s belief in their own abilities [27]. In some other cases, PBC is considered the individual perception of the physical, financial ability and government support to perform some specific behaviors. Through empirical study by Watson (2014), PBC has been proven to have a positive impact on behavioral intention to apply EMA [43]. Yakhou and Dorweiler (2014) argued that a business’s environmental vision and commitment is important and is the beginning of mobilizing efforts and resources for improvements towards corporate sustainability, including perform EMA [48]. Molina et al. (2009) believed that the current management structure and future organizational concepts will determine the ease or difficulty of implementing an innovation [49]. EMA is almost an innovative tool for management accounting in businesses in developing countries, so the institutions of businesses also affect the perception of corporate behavioral control and are closer to the concept of self-efficacy above [27, 43, 48, 49].

*Hypothesis H5*: *Perceived behavior control has a positive impact on the intention to adopt EMA*.

#### Barrier and task complexity (BTC)

Uncertainty theory proposes that task complexity or difficulty has a significant influence on the design of an organization’s environmental management accounting system. Latan et al. (2018) indicated that, the more complex the task, the more difficult it is to perform EMA [50]. Zaid et al. (2018) also found a negative influence of the task complexity factor on the implementation of EMA [51]. Dibrell et al. (2015) pointed out 5 factors affecting EMA adoption including coercive pressure, normative pressure, environmental awareness, environmental vision and task complexity [52]. Except for the factor "task complexity" which has a negative impact, the remaining 5 factors all have a positive impact on EMA implementation. Thus, in order to promote the EMA, it is necessary to reduce the complexity and barriers in the adoption process. Henri et al. (2021) pointed out the barriers preventing EMA applications include cognitive, financial, information, and technical barriers [53]. Jermsittiparsert et al. (2019), in their study in India showed that strongest factor influencing businesses’ application of EMA is financial barriers [54]. Klassen (2019) indicated that lack of information frameworks makes it difficult to effectively collect, identify and evaluate environmentally relevant data and limit the application of EMA [55].

*Hypothesis H6*: *Barrier and task complexity have a negative impact on the intention to adopt EMA*.

#### Policy mix condition (PMC)

Innovation is the result of complex interactions among various actors, including firms, civil organizations, and the public sector [39, 41]. As to Andon et al. (2015), successful innovation depends upon the coevolution of institutions and innovation policies [41]. According to Kwakye et al. (2018), the characteristics of the policy mix condition (coherence, coherence, reliability, comprehensiveness) are important factors promoting the implementation of EMA [45]. The policy mix also includes tools promoted by private actors such as sustainability standards and codes of conduct. Some authors show that this type of tool might have either positive or negative impacts on promoting EMA [4, 18, 38, 39]. Empirical evidence also suggests that firms are more likely to adopt EMA when the policy mix is characterized by a balanced mix of instruments. It is difficult for companies to interact with the EMA without economic incentives, appropriate regulation, tax breaks or some market incentives (e.g., sales and market share expansion) [4, 44, 45]. To promote EMA, policymakers in some countries are adopting an "innovation systems" approach, which sees innovation as the result of complex interactions between all actors, policies and innovation institutions. On the one hand, framework conditions for innovation must be improved, such as the business environment, access to finance, technical advice and trade openness. On the other hand, countries also need targeted innovation policies aimed at both innovation actors and the linkages between them, such as through collaborative projects, public-private partnerships and other clusters [47, 48].

*Hypothesis H7*: *Policy mix condition has a positive impact on the intention to adopt EMA*

#### Firms’ characteristics

Many studies have proven that industry features are critical factors affecting EMA implementation [4, 56, 57]. According to Johnstone (2018), EMA applications appeared more in environmentally sensitive industries such as mining, fertilizer, chemicals, and coal to meet internal management requirements of ministries and environmental concerns of society. In addition, business size can also be a factor having significant influence on EMA. Large-scale enterprises are often subject to more public pressure and political scrutiny [57]. Therefore, these enterprises need to implement EMA to create a good social image and meet the pressure of the community, investors, and the government. Furthermore, large enterprises have higher resources and can apply more sophisticated management accounting techniques than small enterprises. Research by Le et al. (2019) focuses on clarifying the influence of business characteristics on the level of applying EMA. Applying uncertainty theory and using factor analysis and testing techniques, the study showed that large-scale enterprises have a higher level of applying EMA than small-scale enterprises and the level of application of state-owned enterprises is higher than that of private enterprises [58]. Also focusing on clarifying the influence of internal factors on the application of EMA, Latan et al. (2018) pointed out that factors include environmental strategy, managers’ awareness of environmental issues and the commitment of the company manager having positive impacts on EMA adoption by enterprises listed on the Indonesian stock market [50]. In addition, the structure and connection of the accounting department with other parts of the business can also influence EMA adoption behavior. A comprehensive environmental management system requires the participation of relevant departments, including connection between accountants, managers, environmental staff and other functional personnel. The wider the network of expertise, the more likely EMA is to be adopted [4, 18, 55, 56].

*Hypothesis H8*: *Firms’ characteristics have impacts on the intention to adopt EMA*.

**Fig 1:**
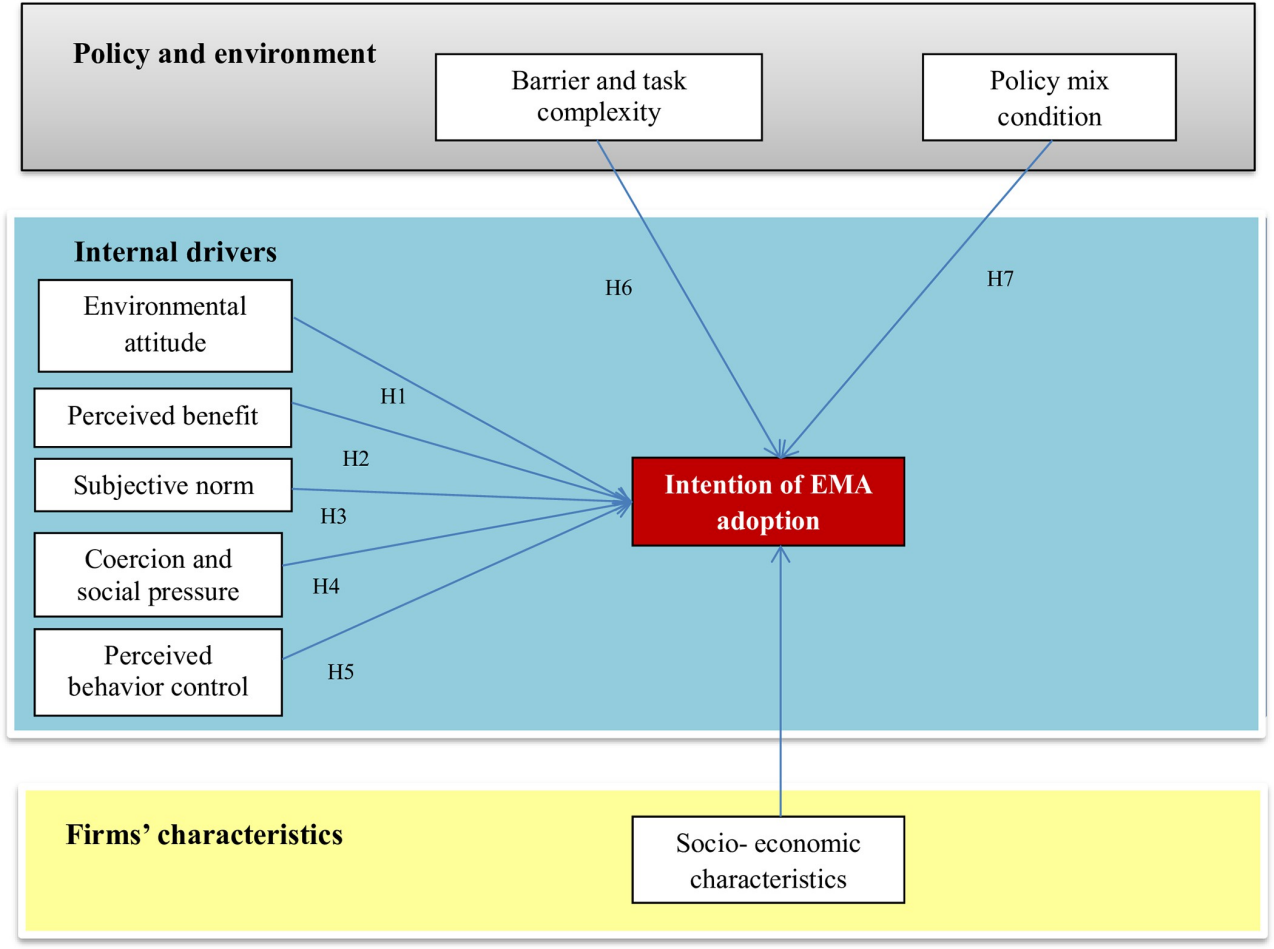
Proposed analytical model of impacted factors of intention to adopt EMA. Source: Research design (2023).

Fig 1 showed the proposed model of this study.

## 3. Data collection and analysis

**Fig 2:**
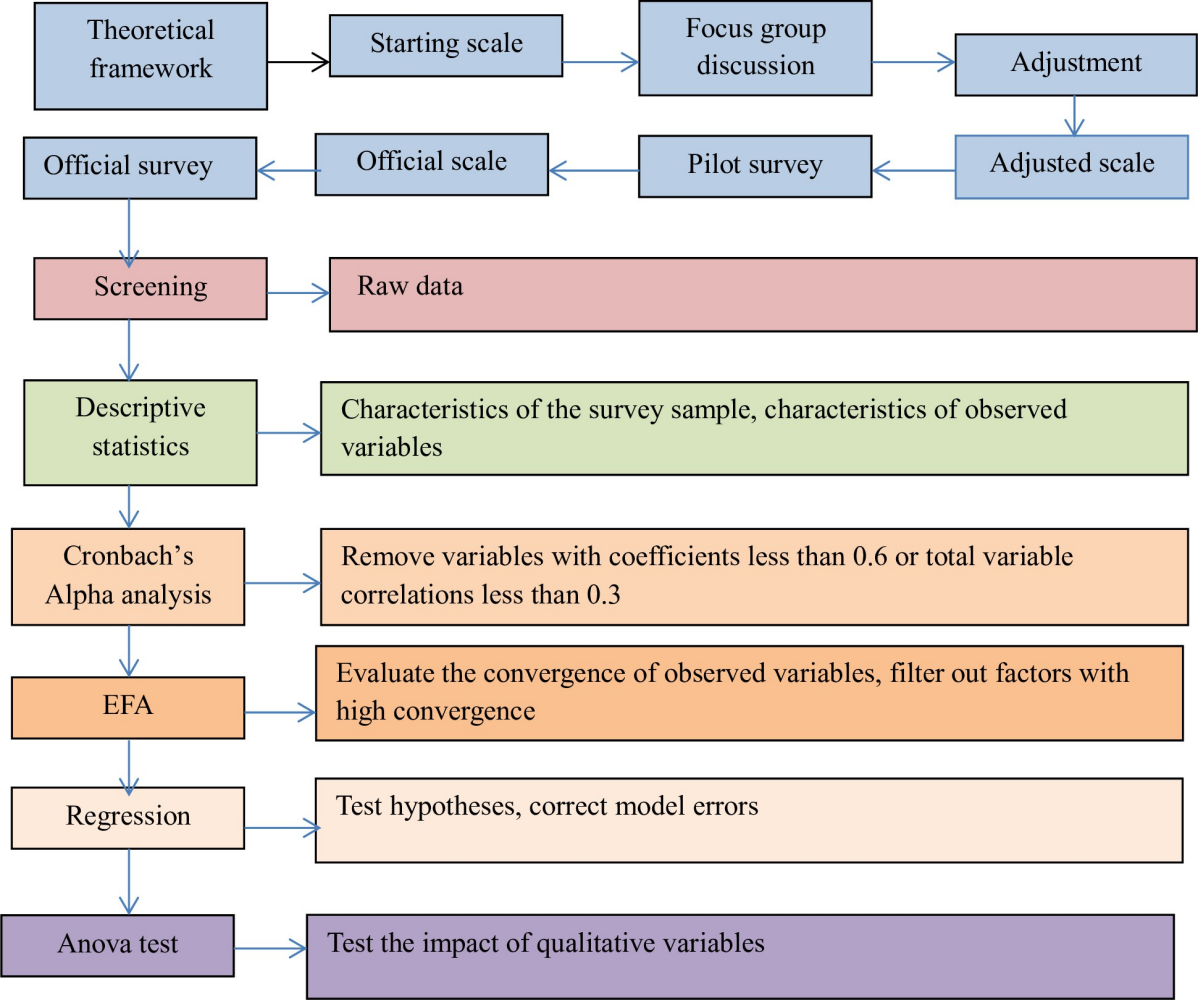
Research process. Source: Research design (2023).

The data collection and analysis process of the study is described in Fig 2.

### 3.1 Data collection

We conducted three stages in the research data collection process. In phase 1, based on the theoretical framework and literature review, expected impact factors were identified along with corresponding scales inherited from previous studies. With 7 expected factors and 1 dependent variable, we initially developed 33 observation variables to measure aspects of these factors. Then a group discussion (FGD) with 9 respondents was conducted. The purpose of FGD was to validate factors, observed variables and scale. FDG was also a forum for respondents to discuss the current situation, challenges and proposals to promote EMA application. Respondents included leaders, chief accountants and accountants of three groups of small, medium and large sized enterprises in the textile industry in Vietnam. From the results of FGD, we added 4 constructs to the measurement group including 1 construct with environmental attitude factor, 1 construct with social pressure factor, 1 construct with subjective norm factor and 1 construct with policy mix condition factor. After adjusting the scales, the study conducted pilot interviews with 10 businesses to validate the questions and complete the interview process (5 businesses belong to the group of potential pollution industries and 5 belong to other industries). After the test, the official questionnaire was completed and had 4 main parts including (i) general information about the business (size, location, industry, number of employees, etc.), (ii) external factors that can influence the firm’s application of EMA include environmental attitude, perceived benefits of EMA, subjective norms, behavioral control and task complexity (iii) external factors that can affect the adoption of EMA include policy mix conditions, coercion and social pressure, and (iv) proposals to promote EMA adoption by businesses.

The study used the following formula to calculate sample size to ensure reliability [59]:

$$n=Z^{2}*\frac{p*(1-p)}{e^{2}}$$

of which n is sample size, *z* is z-score, *e* is margin of error, *p* is standard of deviation. Supposed with a 90% confidence level (z-score as 1.65), 60% standard of deviation and a 5% margin of error, the calculated sample to ensure reliability was 309. In fact, the study collected 330 questionnaires in the official survey by face-to-face interviews.

The study applied cluster sampling combined with random selection to select businesses to participate in interviews. There were two main clusters: size and industry of the business. In terms of size, businesses were divided into 3 groups: large, medium and small enterprises according to the criteria of the Vietnam Enterprise Law (2015) (for example, larger than 200 workers are large-scale enterprises). Industries were divided into two groups: industries with potential to cause pollution and other groups (according to the Law on Environmental Protection 2020). Potential pollution industries included iron and steel, chemicals, paper, textiles, leather and electronics. From the sample of 330, each group divided by industry and size included 55 enterprises (Table 1).

**Table 1:** Selection of businesses by size and industry for survey.

	Large scale	Medium scale	Small scale	Total
Potential pollution industries	55	55	55	165
Other industries	55	55	55	165
Total	110	110	110	330

Source: Research design (2023)

To select specific businesses for the survey, we collected a list of businesses by size and industry from the Business Survey Data Set 2020 (BSDS) by General Statistics Office (GSO). The study selected 4 provinces in Northern Vietnam with many industrial parks and diverse types of industries including Hanoi, Bac Ninh, Vinh Phuc and Phu Tho to conduct interviews. Enterprises according to the list provided by GSO in the above 4 provinces were randomly selected. Before interviewing, the research team contacted businesses by phone and stated the purpose of the interview. If a business refuses, another business was randomly drawn from the list for interview. In the survey, all participants had to give written informed consent to participate in the study. Before answering the questions, the respondents were specifically introduced to the objectives of the interview, the purpose of the research and that the information provided will be confidential and only served for research purposes. At the same time, the respondents were asked if they agreed to participate in the interview. If they agree, they will tick the consent box (agree to participate in the survey) in the consent form, give name and sign the forms with dates of the interviews. In this study, all respondents agreed to participate and ticked into the box of confirmation to participate, gave names and sign in the consent forms with dates of the interview.

The official survey was conducted from 10^th^ May to 10^th^ August 2023. There were 330 questionnaires collected in this survey for analysis.

### 3.2 Instrument development and data analysis

In this study, each impact factor was measured by many observed variables inherited from previous studies. Multidimensional psychological factors such as awareness, attitude or intention cannot be measured by a single observed variable but must be measured by different variables [26, 27]. Accordingly, we developed 5 observed variables to measure each factor: environmental attitude, coercion and social pressure, and perceived behavior control according to studies by Bhattacharyya (2011), Le et al. (2019). Shah and Mohamed (2011), Schaltegger et al. (2012) developed a scale consisting of 4 observed variables to measure the factors subjective norms, task complexity and policy mix conditions. This study also inherited that scale for the case of EMA in Vietnam. However, we adjusted a few small details to better suit the analysis context, for example adding the observed variable about EMA communication to subjective norms. With the variable of perceived benefits of EMA, the study inherited the scale of Watson et al. (2014) and added an additional observed variable (benefit from better connection with investors when applying EMA). Similarly, the variable intention to apply EMA was inherited from the study of Kwakye et al (2018) but added the necessity of applying EMA in the statement of intention by respondents.

The questionnaire included closed questions about observed variables for each potential impact factor. The initial 37 constructs were measured on 5 points Likert scale as previous studies [4, 9, 18, 22, 32]. The respondents were asked to show level of agreement with five statements corresponding to scores from 1 to 5 (1 = “completely disagree, 5 = “completely agree). Table 1 presents the factors, constructs, scale statements and literature sources (Table 2).

**Table 2:** Variables in analytical model.

Factors	Constructs
Environmental attitude (EA)	EA1: I think environmental problems are tending to become more serious in Vietnam and around the world
	EA2: I think business activities are closely related to environmental issues
	EA3: I think businesses must protect the environment
	EA4: I think solving environmental issues in production and operations will make businesses operate more efficiently and sustainably
	EA5: I think environmental protection is an inevitable trend for businesses today
Perceived benefit (PB)	PB1: I think application of EMA helps achieve effective corporate governance
	PB2: I think application of EMA helps firm increase control and reduce risks
	PB3: I think application of EMA helps firm meet customers’ expectation
	PB4: I think application of EMA helps firm use resources more efficiently
	PB5: I think application of EMA helps business improve their social image
	PB6: I think application of EMA helps business connect better with investors
Subjective norm (SN)	SN1: I think company’s core values drives EMA adoption
	SN2: I think the effectiveness of other businesses in applying EMA affects EMA implementation of my firm
	SN3: I think the communication about EMA impacts EMA application
	SN4: I think the trend of innovating management tools impacts EMA adoption
Coercion and social pressure (CSP)	CSP1: I think legal regulations promote the application of EMA
	CSP2: I think market requirements drive EMA adoption
	CSP3: I think industries’ codes of conduct impact EMA implementation
	CS4: I think imitating competitors will drive EMA adoption
	CS5: I think the trend of international economic integration promotes the application of EMA
Perceived behavior control (PBC)	PBC1: I think the degree of compatibility of the internal accounting system with EMA affects the application of EMA
	PBC2: I think the commitment and motivation of business leaders affects the application of EMA
	PBC3: I suspect that businesses’ financial resources are the driving factor in applying EMA
	PBC4: I think the connection between departments in the business affects the application of EMA
	PBC5: I think the responsiveness of an enterprise’s information system drives the adoption of EMA
Barrier and task complexity (BTC)	BTC1: I think that the lack of environmental vision and strategy of businesses will affect the application of EMA
	BTC2: think that organizational limitations of businesses will affect the application of EMA
	BTC3: I think that businesses’ lack of finance is a barrier to implementing EMA
	BTC 4: I think lack of information is a barrier to implementing EMA
Policy mix condition (PMC)	PMC1: I think the comprehensiveness of the state’s policy promotes the application of EMA
	PMC2: I think the clarity and coherence of the state’s policies promotes the application of EMA
	PMC3: I think the commitments and stability of state’s policies promote the application of EMA
	PMC4: I think that the State’s business support policies promote the application of EMA
Intention to adopt EMA (IEMA)	EMA1: I will adopt EMA in the future
	IEMA2: Applying EMA is my priority in perfecting the corporate management accounting system
	IEMA3: I will apply EMA with supports from management agencies
	IEMA4: Implementing EMA with businesses is inevitable

Source: Research design (2023)

Survey data was analyzed by SPSS 23.0 version. First, data was cleaned, coded and checked for consistency before statistical analysis procedures. There were 15 survey questionnaires with missing data, so they were eliminated and there were only 315 valid questionnaires left for analysis. Descriptive statistics (frequencies, percentages, means, and standard deviation), chi-square tests for 2-way categorical correlations, and t-tests for continuous variables were then performed. Next, Cronbach’s Alpha analysis was undertaken to test reliability of the scales. Cronbach’s Alpha test reflected the degree of correlation between observed variables in the same factor. More specifically, it showed which of the observed variables of a factor contribute to the measurement of that factor concept. Accordingly, any observed variables with Cronbach’s Alpha coefficient less than 0.6 or total variable correlation less than 0.3 were eliminated to retain sufficiently reliable variables [59]. Next, EFA was conducted to evaluate the convergence of observed variables on the factors. In particular, EFA was significant when factor loading > 0.5, Kaiser-Meyer-Olkin (KMO) coefficient ranged from 0.5 to 1, Bartlett’s test was statistically significant (Sig. < 0.05), and percentage of variance > 50%. The valid factors after EFA then were used for regression analysis to identify factors affecting intention of EMA adoption. To test the reliability of regression model, we used F statistic, along with checking for issues such as multicollinearity, autocorrelation, and unequal variance. The Anova test is used to evaluate the impact of business characteristics variables on the intention to apply EMA. The equation of factors affecting the intention to implement EMA by firms was as follows:

$${IEMA}_{i}=\alpha +\beta_{1}*EA+\beta_{2}*PB+\beta_{3}*SN+\beta_{4}*CSP+\beta_{5}*PBC+\beta_{6}*BTC+\beta_{7}*PMC+e_{i}$$

of which α is intercept, β_i_ coefficient of impact factors, *e_i_* is error.

## 4. Results

### 4.1 Sample characteristics

In the 315 valid questionnaires, 54.3% of respondents were male and 45.7% of respondents were female. Regarding work positions, 57% of respondents belonged to firms’ boards of management and administrative councils, 43% were chief accountants and accountants. These people have a role in orienting, designing and implementing management accounting and EMA In terms of industries, the survey was conducted with businesses in polluting industries such as iron and steel, chemicals, paper, textiles (47.3%) and other industries (52.7%). There were 125 state-owned enterprises (39.7%) and 190 private enterprises (60.3%) interviewed. It can be seen that the research sample covered aspects of businesses including size, industry, and ownership type in Vietnam (Table 3).

**Table 3:** Sample characteristics.

Characteristics		Number	Percentage (%)
Gender	Male	171	54.3
	Female	144	45.7
Workplace	Board of management	123	39.0
	Administrative council	46	14.6
	Chief accountant	57	18.1
	Accountant	89	28.3
Sector	Iron and steel	23	7.3
	Chemistry	34	10.8
	Paper production	16	5.1
	Leather, shoes, textiles	55	17.5
	Electronic	21	6.6
	Other industries	166	52.7
Business owner	State	125	39.7
	Private	190	60.3

Source: Research results (2023)

### 4.2 Cronbach’s Alpha analysis

**Fig 3:**
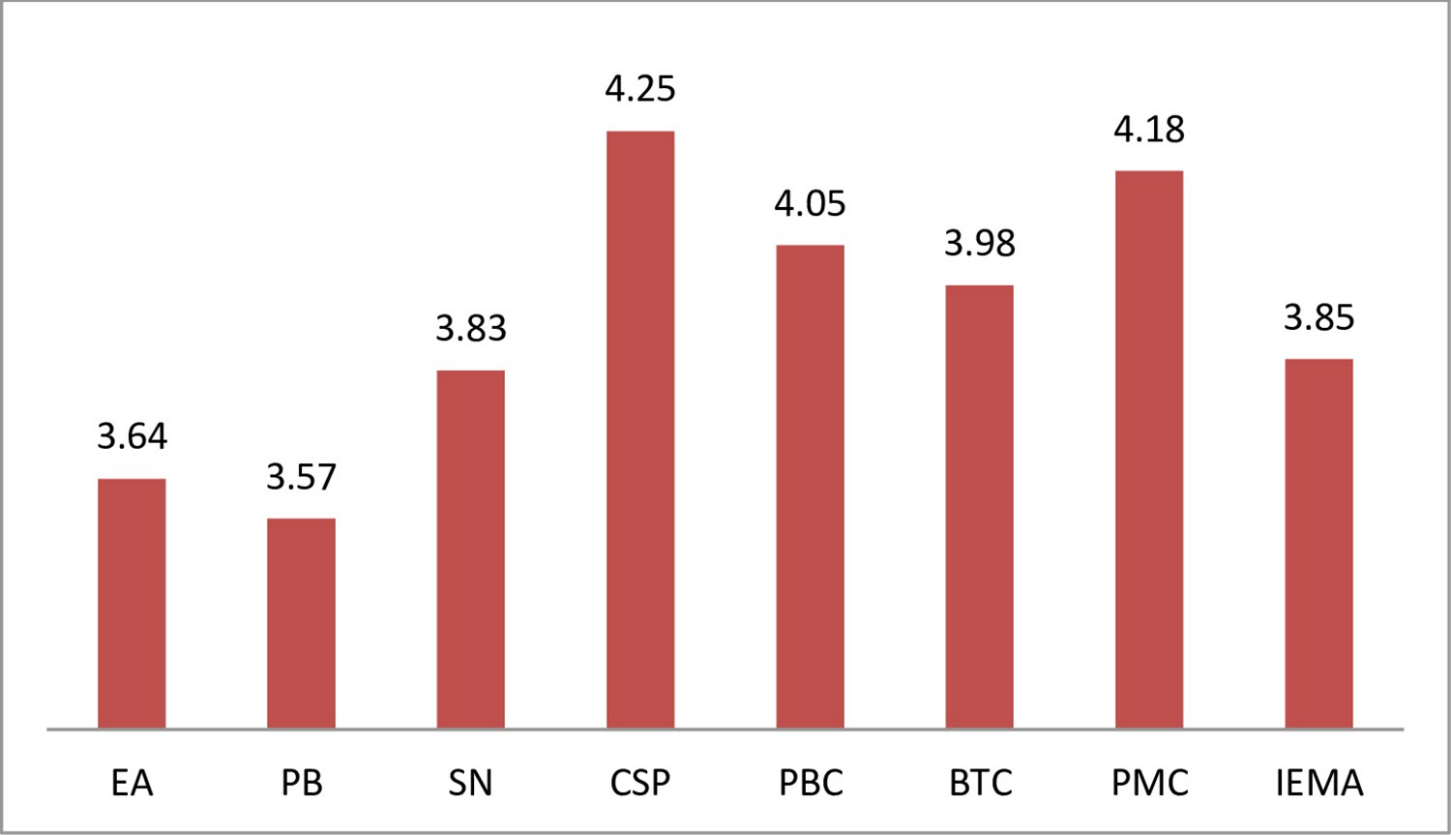
Means of scales by factors. Source: Research results (2023).

The reliability of the measurement scale was assessed using Cronbach’s Alpha analysis based on the quantitative aspects in the questionnaires. The analysis results showed that all 37 measurement scales had reliable values for their respective factor groups (Cronbach’s alpha coefficients >0.6 and corrected item—total correlation > 0.3). The CSP variable has the highest Cronbach’ Alpha coefficient (0.901), and the PMC variable had the lowest Cronbach’ Alpha coefficient (0.841). The value of constructs ranged from 1 to 5 points depending on factors. In which the average value of the CSP factor was the highest (4.25 points) and the PB factor was the lowest (3.57 points) (Fig 3). This demonstrated that the research concepts built from the observed variables achieved internal consistency, all constructs contribute well to measuring the relevant factor and ensured reliable measurements [59]. All constructs were hence used for EFA (Table 4).

**Table 4:** Results of the reliability testing of the measurement scale.

Factors	Cronbach’s Alpha coefficients	Corrected item—total correlation	Range of scale (points)
EA	0.848	0.793–0.836	3–5
PB	0.882	0.801–0.874	1–5
SN	0.852	0.798–0.843	2–5
CSP	0.901	0.600–0.775	3–5
PBC	0.863	0.721–0.831	2–5
BTC	0.848	0.765–0.823	1–5
PMC	0.841	0.805–0.831	1–5
IEMA	0.845	0.771–0.824	2–5

Source: Research results (2023)

### 4.3 Exploratory factor analysis

EFA was conducted on 33 observed variables belonging to 6 independent factors and 4 observed variables belonging to dependent variables. Hair et al. (2013) suggested that combining independent and dependent variables in the same EFA for examining the scale converges was inappropriate. The extraction method used was Principal Component Axis (PCA) which reflected the data structure more accurately as well as being suitable for analyzing the multiple regression models [59].

Table 5 showed EFA results of independent variables. In the first round of EFA, 3 constructs including PBC2, PBC4 and BTC2 had factor loading values < 0.3 so were eliminated. In the second round of EFA, the factor loading values of the remaining 30 constructs all had values greater than 0.7, so they were valid, and no observed variables were eliminated. The KMO was 0.899 (> 0.5), and the Bartlett’s test had a significant value (Sig = 0.00) indicating the appropriateness of EFA model. The analysis revealed that at Eigenvalues > = 1 and 6 factors were extracted from the 30 observed variables accounting for 75.16% of the total extracted variance. In which the PBC and BTC factors had converted into a new factor and was renamed as ‘perceived control and barrier’ (PCB). The 5 factors including EA, PB, SN, CSP and PMC remained the same as before.

**Table 5:** Results of EFA for independent variables.

Observed variables	Factors					
	1	2	3	4	5	6
EA1	0.914					
EA3	0.902					
EA4	0.987					
EA5	0.871					
EA2	0.863					
PB2		0.912				
PB4		0.897				
PB3		0.872				
PB1		0.844				
PB6		0.827				
PB5		0.801				
SN2			0.876			
SN4			0.823			
SN3			0.787			
SN1			0.764			
CSP4				0.855		
CSP1				0.814		
CSP2				0.812		
CSP3				0.764		
CSP5				0.726		
PBC2					0.911	
BTC1					0.891	
PBC3					0.875	
PBC5					0.856	
BTC3					0.814	
BTC4					0.799	
PMC2						0.841
PMC1						0.789
PMC3						0.767
PMC4						0.752
KMO	0.891					
Eigenvalue	1.028					
Sig Bartlett	0.000					
Total Variance Extracted	75.160					

Source: Research results (2023)

Next, EFA analysis of the dependent variable (IEMA) showed that coefficient KMO = 0.730 > 0.5, Bartlett’s test had Sig. = 0.000 < 0.05, the Eigenvalue of the first factor was 3.255 > 1 showing the convergence of the analysis stopping at the first factor, the total variance extracted was 74.25% > 50% of the loading factor. All factor loading values were > 0.5. Thus, there was 1 factor extracted from the 4 observed variables for the intention to apply EMA, and this factor explained up to 74.25% of the variation in the data set. IEMA thus was the valid factor for dependent variable and later regression analysis.

### 4.4 Regression analysis

To test the hypotheses and identify factors affecting the intention to implement EMA in Vietnamese enterprises, we ran multiple regression analysis based on factors after EFA. Tables 6 and 7 presented detailed results of the regression model. The results showed that Sig of F test = 0.000 < 0.05, proving that the selected model fitted data set. With an adjusted R^2^ value of 0.712, it implied that 71.2% of the variance in dependent variable was explained by independent variables. The VIF coefficients of the independent variables are all less than 2.0, proving there was no multicollinearity. In addition, the Durbin—Watson value of 1.805 was between 1.5 and 2.5, indicating that autocorrelation did not occur in the regression [59].

**Table 6:** Model summary.

R	R^2^	Adj R^2^						Durbin Watson
			Std error of the estimate	F	Df1	Df2	Sig F.	
0.841	0.743	0.712	0.563	48.134	6	0.315	0.000	1.805

Source: Research results (2023)

**Table 7:** Regression results.

Model	Unstandardized coefficients		Standardized coefficients	t	Sig.	Multicollinearity	
	B	Std. Error	Beta			Tolerance	VIF
Constant							
EA	0.212**	0.041	0.202**	2.863	0.015	0.752	1.330
PB	0.185***	0.061	0.179***	1.384	0.000	0.890	1.123
SN	0.120**	0.041	0.103**	2.863	0.023	0.752	1.308
CSP	0.223***	0.055	0.207***	1.117	0.000	0.653	1.532
PCB	0.147**	0.036	0.132**	2.003	0.031	0.735	1.460
PMC	0.176**	0.044	0.171**	2.102	0.028	0.679	1.635

Note

***, ** correspond for the significant level of 1% and 5%.

Source: Research results (2023)

The regression results indicated, all 6 independent variables had significant relationship with the intention to apply EMA in Vietnamese enterprises in which CSP was the variable with the strongest impact and SN was the variable with the weakest impact. The standardized regression model was as following:

IEMA=0.246+0.207*CSP+0.202*EA+0.179*PB+0.171*PMC+0.132*PCB+0.103*SN

To evaluate the impact of business characteristics variables on the intention to apply EMA, the study used ANOVA test with EIMA score as the dependent variable and business characteristics including industry, size, and ownership type are independent variables. Regarding the industry of the business, the results showed that the average IEMA points of industries with polluting potential was significantly higher than that of other industries. Thus, potentially polluting industries have greater intention to implement EMA than other industries. There were also significant differences between businesses of different sizes in their intention to implement EMA. Tamhane’s pairwise variance test indicated that there was a significant difference in intention to adopt EMA between large and medium sized enterprises, between large and small sized enterprises, but no difference was found between enterprises medium-sized to small-sized businesses. For ownership type, ANOVA analysis showed that Sig. = 0.062 > 0.05 which means there was no difference between the intention to apply EMA of state-owned enterprises and private enterprises (Table 8).

**Table 8:** Anova results of mean difference by gender.

Factors	Groups	Mean of IEMA scores	Std.deviation	Sig.
Industries	Potential pollution	3.847	0.346	0.018
	Others	3.208	0.423	
Scale	Large size	3.903	0.358	0.033
	Medium size	3.351	0.303	
	Small size	3.223	0.408	
Owner	State	3.637	0.242	0.062
	Private	3.572	0.369	

Source: Research results (2023)

**Fig 4:**
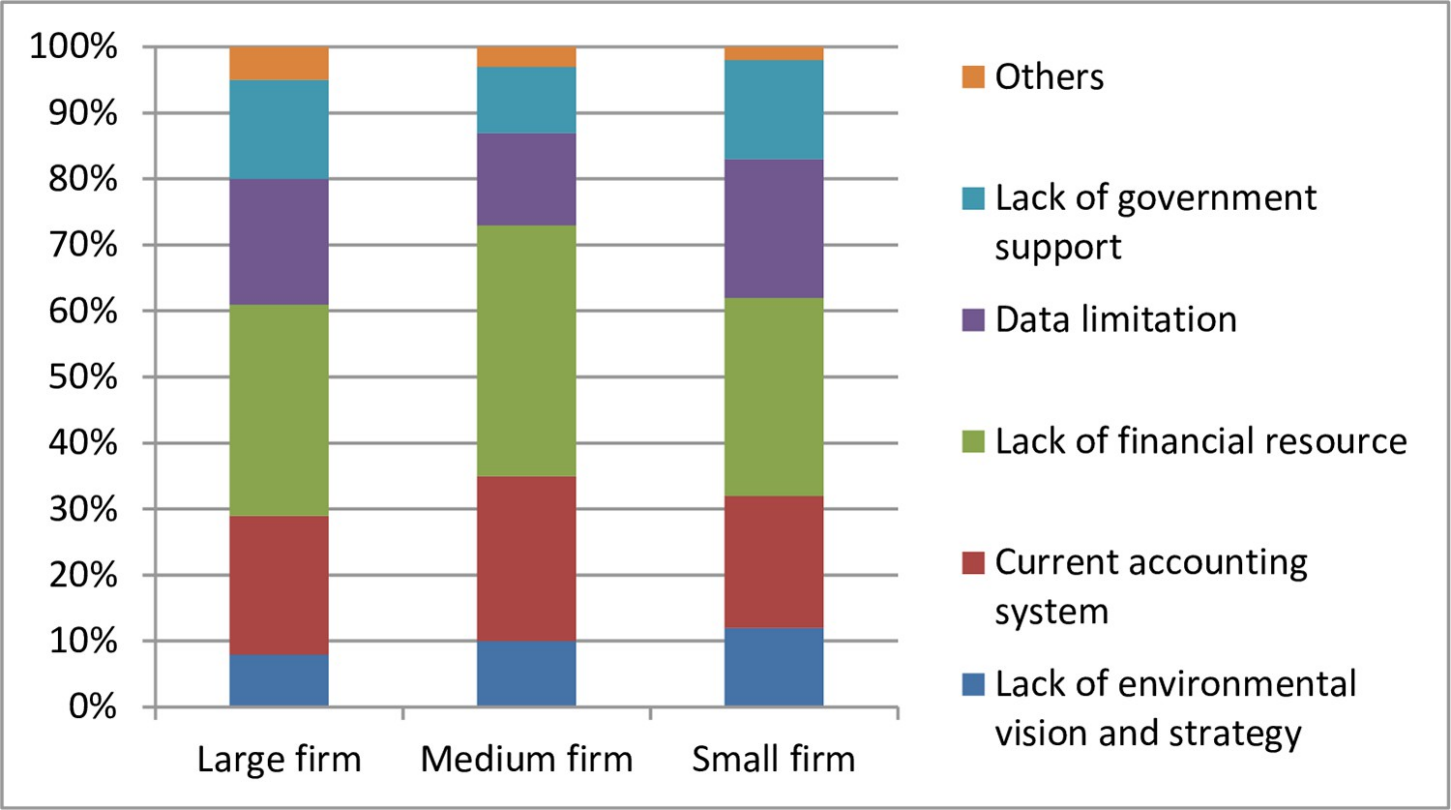
Challenges in EMA adoption intention. Source: Research results (2023).

The survey also identified difficulties that businesses encountered in their intention to implement EMA. The results showed that there is a similarity among all three groups of businesses (large, medium, small) that lack of finance and government support was the biggest challenges to implementing EMA. However, the difference was that small and medium-sized enterprises emphasize data challenges while large enterprises emphasize the difficulties of the enterprise’s environmental vision and commitment. Large and medium-sized enterprises also believed that the current accounting system is not compatible with EMA and that if they want to implement EMA, they need to edit and supplement the internal management accounting system. Small businesses also saw this as a challenge, but not a big one, because they think that with a small scale, adjusting the accounting system is more compact and easier (Fig 4).

## 5. Discussions

This article evaluated how factors affecting intention of EMA adoption in Vietnamese firms. We tested several models to examine the impact of behavioral predictors, firm characteristics, and policy mix conditions on EMA adoption. The results demonstrated that EMA applying intention was not solely the result of firm behavioral predictors (e.g., perceived benefits and environmental attitude) but also significantly determined by the policy environment.

Firstly, ‘coercion and social pressure’ is the factor that most strongly influences businesses’ intention to implement EMA. CSP includes pressures from the government, the market and competitors and has a positive influence on business behavior in applying EMA. This result is consistent with many previous studies of Bhattacharyya (2011), Yakhou and Dorweiler (2014), Olaoye and Olaipekun (2018). In these studies, social pressure is divided into coercive pressure, normative pressure and mimetic pressure. In particular, coercive pressure is expressed through government regulations, fines, and pressure from shareholders, local governments and environmental organizations. Normative pressure is expressed through industry standards, while imitative pressure is the pressure from competitors, other industries in the same area and multinational organizations. The results of this study showed that government regulations have a strong influence on the design and operation of corporate accounting systems. If the government has regulations and requirements on EMA, this is an important motivation for businesses to implement EMA to comply with the requirements. Of course, implementing EMA cannot be done immediately but requires a process. In particular, in addition to mandatory requirements, the government needs to have a vision and orientation along with incentives and support in the early stages so that businesses have a better awareness of EMA, thereby gradually applying EMA. An also provides recommendations on the role of government in the early stages of promoting EMA in developing countries. With the pressure of the market and competitors, Vietnamese businesses also believed that they are greatly affected by these factors, especially exporting businesses, which must comply with environmental regulations and standards. They believed that applying EMA is necessary and should be done early to help enhance the social image of businesses as well as better satisfy the requirements of the market, customers and society. This has also been discovered in studies of Xiaomei (2014) in China, Olaoye and Olaipekun (2018), and Maama and Appiah (2019) in Europe.

The second most influential factor in the intention to apply EMA is ‘environmental attitude’. According to TPB theory, attitude is the first and important part that affects attitudes and behavior. Attitude is related to a business’s perception and perception of a certain issue or behavior [26, 28]. In this study, environmental attitudes related to businesses’ perception of the relationship between production and the environment, the need for businesses to participate in environmental protection, and the tendency of businesses to manage environmental management to increase business efficiency. The results showed that attitude has a positive influence on the intention to perform EMA behavior. This is similar to the results in studies by Al (2013), Modell (2014), Tarkiainen et al. (2015), and many other studies on corporate behavior [33–35]. According to Maama and Appiah (2019), attitude is largely determined by awareness. In the case of EMA, the change is a shift from an old state to a new state due to the perceived future benefits of EMA. Therefore, increasing awareness of business owners or chief accountants about EMA is indispensable to help change attitudes about applying EMA. In Vietnam, according to culture, the leadership hierarchy is very clear, so the leadership role of the leader is very important for changing organizational behavior. If leaders have awareness and vision of sustainable development, they will be interested in corporate environmental management and have a higher ability to apply EMA. In addition to leadership and support from leaders, knowledge sharing and training also play an important role in EMA application. EMA is a difficult job and requires information from many different departments, so it requires mutual sharing and learning between departments, especially between the accounting and environmental management departments. These implications were also mentioned in the studies of Maama and Appiah (2019).

‘Policy mix condition’ is the third strongest impact factor of EMA adoption. In fact, the government’s support through policies always plays an essential role in the behavior of businesses, including EMA. The stronger the support from government, the higher likelihood of EMA adoption by businesses. Supporting policies need not only unity but also harmony and trust. Commitment also plays an important role and guides business behavior. Supportive policies that create economic incentives would be easier for businesses to apply than command-and-control policies. This result is consistent with the importance of issuing clear and consistent regulations on EMA. Information and technical support from regulatory agencies are also considered essential for businesses to reform their management accounting systems and integrate environmental aspects in this system. Integration must ensure consistency and efficiency for businesses, so they need more specific and systematic instructions from the government. A study on EMA behavior in Malaysia also pointed out that regulations and guidelines for EMA implementation will determine the success of a business’s EMA organization and design [27]. In addition to national supporting policies, industry policy also plays a key role in the application of EMA. This research showed that industry standards, implementation principles or industry development strategies have a positive impact on EMA behavior. Therefore, the support policy framework should be improved at both national and sector-specific levels. These findings are also demonstrated by studies by Johnstone (2018), Roy and Ghosh (2019).

The results also indicated that ‘perceived benefits’ positively and significantly affect behavioral intention to apply EMA. EMA not only helps businesses save on operating costs but also contributes to making them more efficient and sustainable. Other studies around the world such as Schaltegger et al. (2012), Wild and Van Staden (2012), Vejzagi et al. (2018) also have similar results about the positive impact of PBC on EMA application. Therefore, if the Vietnamese government wants to apply EMA effectively and successfully, it needs to widely propagate its benefits through seminars and training on EMA for businesses; moreover, further improving the legal corridor so that businesses have a basis to develop EMA. For businesses that are already aware of the benefits of EMA, it is necessary to research and prepare resources to apply EMA effectively.

Factor ‘perceived control and barrier’ also has a positive impact on the intention of adopting EMA in Vietnam firms. This is consistent with the TPB theory that increased perceived control leads to increased behavioral intention [26]. Research results showed that the application of EMA can be limited by internal business barriers such as business owners’ low attitude towards the environment, financial shortages, technical difficulties or lack of compliance of the current accounting system with EMA. Thus, compared to the research of Shah and Mohamed (2011), this study has shown that, in addition to external factors and especially institutional pressure (law and regulations), the application of EMA is also influenced by other factors within the firm such as the attitude, awareness of administrators and financial capacity of the unit. To promote the application of EMA, it is necessary to gradually eliminate these barriers, in which the government and industry associations have an important supporting role as shown by Molina et al. (2009), Watson (2014), Yakhou and Dorweiler (2014).

In this study, ‘subjective norm’ is the factor that has the weakest influence on businesses’ intention to apply EMA. Norms include the internal core values of the business, codes of conduct in the industry, EMA trends of the business community and impacts from the media. As the norm for sustainable development increases, the likelihood of applying EMA also increases. This result is consistent with TPB theory on the role of norms and studies by Cordeiro and Sarkis (1997) and Setthasakko (2017) on the impact of subjective norms on businesses’ intention to implement EMA. Currently, large enterprises mainly in the foreign direct investment (FDI) sector have environmental visions and strategies, in which they declare core values including sustainable development and environmental protection. However, most small and medium-sized Vietnamese enterprises do not have environmental vision and commitment. The role of codes of conduct in the industry is then very important to promote EMA adoption. Tarkiainen et al. (2015) also pointed out that in developing nations, businesses often do not develop core environmental values. Therefore, to promote friendly behaviors, pressure from society, government and especially relevant industry associations must be increased. The practice principles of some industries in Vietnam also encourage the application of EMA to increase environmental management efficiency and social responsibility connection with customers. This factor recommends to the Vietnamese government on creating a favorable environment for EMA development, including requirements for industries or business groups to apply EMA. EMA can start applying to industries with high pollution potential, and then expand to other industries according to a specific roadmap.

Finally, ‘characteristics of business,’ specifically sector and scale also have significant impact on EMA applying intention. This is appropriate as different industries are operating under different pressures. Latan et al. (2018) affirmed that the application of EMA really depends on the industry; polluting firms tend to apply higher EMA. This study similarly demonstrated differences between EMA adoption intentions between polluting industries and other industries. In Vietnam, or other developing countries, polluting businesses, despite causing negative impacts on welfare, still operate widely and bring high economic value. These businesses have recently changed their attitude towards environmental protection due to pressure from the market and the countries importing their products. EMA is a tool that can help businesses better manage the environment, thereby reducing social pressure. That is also one of the motivations for businesses that have the potential to cause pollution to better perform their social responsibilities. Similar results were also discovered in studies by Adon et al. (2015), Johnstone (2018), and Le et al. (2019).

## 6. Conclusions

This study draws out factors affecting the intention to apply EMA in Vietnamese enterprises based on theoretical foundation of TRA, TPB and TAM. Analytical results using Cronbach’s Alpha, EFA, regression and ANOVA showed that there were 6 factors driving businesses’ intention including environmental attitude, perceived benefits of EMA, subjective norms, coercion and social pressure, perceived control and barrier, and policy mix condition, in which coercion and social pressure was the strongest influencing factor. Both internal and external environment factors had a meaningful impact on a business’s EMA behavior. In addition, the intention to apply EMA was also determined by the industry and size of the enterprise, in which industries with the potential to cause pollution and large-scale enterprises had higher intentions to apply EMA.

This research has both theoretical and practical contributions. Theoretically, we expanded influencing factors from the original model of TPB. Specifically, from the 3 main components of TPB, the study developed 7 factors and corresponding scales to evaluate specific EMA situations in Vietnam. The study also found that the variable perceived behavioral control can combine with perceptions of barriers to form a new factor (perceived control and barriers). In addition, we also mixed the behavioral psychology model with business characteristic variables to detail the picture of factors affecting EMA behavior. Empirically, the research contributes to proposing a number of management implications.

Firstly, state agencies need to perfect a synchronous system of policies on EMA. Government enforcement is a strong factor leading to changes in corporate behavior. Without coercion, voluntary EMA adoption is very difficult in Vietnam, especially in the initial stages. Policies hence should be designed in both coercive and supportive directions. In fact, the organization of EMA work in Vietnam has not received much attention from businesses because there are no specific documents prescribed by the State. Therefore, the State needs to soon issue regulations on the organization of EMA work, the accounting system used, as well as the accounting and reporting of income and expenses from environmental protection of businesses. Karma. In case of necessity, mandatory sanctions can be applied to ensure the implementation of environmental accounting at enterprises, on the basis of which the State can better control environmental protection work of business.

Secondly, it is necessary to develop a vision and roadmap for applying EMA. The application of EMA can start with polluting industries and large-scale enterprises, then expand to small and medium-sized enterprises. Industry associations also need to issue codes of conduct on EMA and encourage businesses to do so voluntarily in the initial stages, and then mandatory to satisfy the requirements of the associations, market, investors and customers. The state and industry associations should have communication programs, training courses, and capacity building for businesses about EMA. In the short run, the Ministry of Finance needs to consider and gradually draft and promulgate regulations on EMA, guiding EMA tools so that they can be applied at businesses at many levels. In addition, the Ministry of Natural Resource and Environment should also promulgate an environmental reporting system and regulate the publication of periodic environmental reports with a system of indicators built on a scientific basis, complying with international standards and taking into account the specific conditions of firms.

Thirdly, business owners and chief accountants need to better understand the benefits of EMA and as understanding improves, the attitude and behavior of applying EMA might also improve. Business managers need to regularly pay attention to updating and implementing environmental accounting in businesses, especially for manufacturing businesses; Focus on investing financial and human resources in the accounting apparatus, including EMA. In addition, it is necessary to focus on investing financial resources in the accounting apparatus, including EMA. In the early stages, environmental accounting should be tested at one line or one department before mass application to the entire enterprise. Businesses develop long-term business strategies that take into account the impacts of products’ environmental standards and regulations.

This study has some limitations that should be explored in further researches. Firstly, the sample includes businesses that already have or have not applied EMA, so the research only analyzed the impact of factors affecting the intention to implement EMA, not the actual behavior of applying EMA. In addition, the research sample size was small and only focused on the North region of Vietnam with 4 representative provinces. For these reasons, future studies can choose large sample sizes with many regions in Vietnam as well as businesses with EMA adoption to identify factors affecting actual behavior and deeper testing of TPB with EMA case in Vietnam. In addition, future studies can add some other factors in the model to improve the ability to explain the intention and actual behavior of implementing EMA. Last but not least, SEM analysis can also be taken into account in future studies in analyzing layers of influencing factors instead of a linear model with only one layer of impact factors as in this study.

## Supporting information

S1 Data (XLSX)

## References

[1] Bromley DW. Environmental regulations and the problem of sustainability: Moving beyond “market failure”. Ecological Economics. 2007; 63(4): 676–683.

[2] Famiyeh S, Adaku E, Amoako K, Asante D, Amoatey C. Environmental management practices, operational competitiveness and environmental performance: Empirical evidence from a developing country. Journal of Manufacturing Technology Management. 2018; 29(3): 588–607.

[3] Schaltegger S, Synnestvedt T. The link between ‘green’ and economic success: Environmental management as the crucial trigger between environmental and economic performance. Journal of Environmental Management. 2002; 65(4): 339–346. 12369398

[4] Bhattacharyya A. Attitudes towards environmental accountability in an emerging economy setting Evidence from India. J. Asia Pac. Cent. Environ. Account. 2011; 17: 51–74.

[5] Gul F. The effects of management accounting systems and environmental uncertainty on small business managers’ performance. Accounting and Business Research. 1991; 22(85): 57–61.

[6] Roger L, Stefan S. Environmental management accounting and supply chain management, Eco-Efficiency in Industry and Science. 2011; 27: 18–30.

[7] Manrique S. Martí-Ballester CP. Analyzing the effect of corporate environmental performance on corporate financial performance in developed and developing countries. Sustainability. 2017; 9, 1957.

[8] Spence C, Husillos J. Correa-Ruiz C. Cargo cult science and the death of politics: A critical review of social and environmental accounting research. Crit. Perspect. Account. 2010; 21: 76–89.

[9] Xiaomei L. Theory and practice of environmental management accounting. Int. J. Technol. Manag. Sustain. Dev. 2014; 3: 47–59.

[10] Hussain SS. The ethics of “going green”: The corporate social responsibility debate. Business Strategy and the Environment. 1999; 8(4): 203–210.

[11] Reed MS. Stakeholder participation for environmental management: A literature review. Biological Conservation. 2008; 141(10): 2417–2431.

[12] Alkisher AO. Factors influencing environmental management accounting adoption in Oil and manufacturing firms in Lybia. Thesis of doctor, Utara Malaysia University, 2013.

[13] Duc TD, Tho DT, Huy HL. Factors affecting climate-smart agriculture practice adaptation of farming households in coastal central Vietnam: The case of Ninh Thuan province. Front. Sustain. Food Syst. 2022; 6: 790089. doi: 10.3389/fsufs.2022.790089

[14] World Bank. Resilient shores: Vietnam’s coastal development between opportunity and disaster risk. Vietnam. 2020; Vietnam.

[15] Dat TT, Truong DD. Resource management, environment and climate change towards development sustainability in Vietnam from an economic perspective Journal of Economics and Development. 2020; 278(2) 2–11.

[16] Truong DD. Farming households’ satisfaction with quality of agricultural extension services: a case study of Quang Binh province, Vietnam. Front. Sustain. Food Syst. 2022; 5:779477.

[17] Le TNP, Nguyen KH. Impact of removing industrial tariffs under the European–Vietnam free trade agreement: A computable general equilibrium approach, Journal of Economics and Development. 2019; 21(1): 2–17.

[18] Le TT, Nguyen TMA, Phan TTH. Environmental management accounting and performance efficiency in the Vietnamese construction material industry—a managerial implication for sustainable development. Sustainability 2019; 11: 5152.

[19] Bebbington J, Russell S, Thomson I. Accounting and sustainable development: reflections and propositions. Critical Perspectives on Accounting. 2017; 48: 21–34.

[20] Govindarajan V. Appropriateness of accounting data in performance evaluation: An empirical examination of environmental uncertainty as an intervening variable. Accounting, Organizations and Society. 1984; 9(2): 125–135.

[21] Christ B, Environmental management accounting: the significance of contingent variables for adoption. Journal of Cleaner Production. 2013; 41: 163–173.

[22] International Federation of Accountants (IFAC). International guidance document: environmental management accounting. New York, 2005.

[23] United Nations Division of Sustainable Development (UNDSD). Integrated environmental and economic accounting: an operational manual. NewYork, 2000.

[24] Schaltegger S, Petersen H. An introduction to corporate environmental management: Striving for sustainability. Management of Environmental Quality: An International Journal. 2013; 14(4): 541–542.

[25] Fishbein M, Ajzen I. Understanding attitudes and predicting social behavior. Englewood Cliffs Prentice- Hall NJ, 1980.

[26] Ajzen I. The theory of planned behavior: Organizational behavior and human decision processes. 1991; 50(2): 179–211.

[27] Shah AS. Mohamed SN. (2011). Applying the theory of planned behavior (TPB) in halal food purchasing. International journal of Commerce and Management, 21(1), 8–20.

[28] Dima A. Green purchasing behavior: green products and Ajzen’s theory of planned behavior Economic and Behavior. 2013; 34: 45–59.

[29] Davis FD. Technology acceptance model: TAM Information Seeking Behavior and Technology Adoption. 1989; 3: 205–219.

[30] Davis FD. User acceptance of information technology: system characteristics, user perceptions and behavioral impacts International Journal of man-machine studies. 1989; 38 (3): 475–487.

[31] Tarkiainen A, Sundqvist S. Subjective norms, attitudes and intentions of Finnish consumers in buying organic food. Br. Food J. 2015; 107: 808–822.

[32] Al K. Factors influencing environmental management accounting adoption in oil and manufacturing firms in Libya. PhD. Thesis. University Utara Malaysia, 2013.

[33] Jasch C. Environmental management accounting: comparing and linking requirements at micro and macro levels—a practitioner’s view. Environmental Management Accounting and Supply Chain Management. 2011; 5: 255–277.

[34] Roy A, Ghosh SK. Determinants of corporate environmental disclosure from an Asian perspective. IIM Kozhikode Soc. Manag. Rev. 2019; 8: 171–189.

[35] Elisabeth A. Does environmental management improve financial performance? A meta-analytical review. Organization and Environment. 2013; 26(4): 431–457.

[36] Maama H, Appiah KO. Green accounting practices: Lesson from an emerging economy. Qual. Res. Financ. Mark. 2019; 11, 456–478.

[37] Modell S. The societal relevance of management accounting: An introduction to the special issue. Account Bus. Res. 2014; 44: 83–103.

[38] Schaltegger S. Viere T, Zvezdov D. Tapping environmental accounting potentials of beer brewing: Information needs for successful cleaner production. J. Clean. Prod. 2012; 30: 1–10.

[39] Wild S, Van Staden C. Integrated reporting: Initial analysis of early reporters—An institutional theory approach. In Proceedings of the 7th Asia Pacific Interdisciplinary Accounting Research Conference, Kobe, Japan, 2013; 26–28.

[40] Vejzagi V, Brown JB. Schmidt P. Accounting for sustainability: environmental indicators from Croatian hotels. Int. J. Bus. Manag. Commer. 2018; 3: 24–34.

[41] Andon P.; Baxter J.; Chua W.F. Accounting for Stakeholders and Making Accounting Useful. J. Manag. Stud. 2015, 52, 986–1002.

[42] Yusoff H, Ismail A, Othman R. Motives and accountants’ role for green accounting-reporting towards minimizing financial leakages. Manag. Account. Rev. 2016; 15: 35–55.

[43] Watson K, Klingenberg B, Polito T, Geurts TG. Impact of environmental management system implementation on financial performance: A comparison of two corporate strategies. Management of Environmental Quality: An International Journal. 2014; 15(6): 622–628.

[44] Cordeiro J, Sarkis J. Environmental proactivism and firm performance: Evidence from security analyst earnings forecasts. Business Strategy and the Environment. 1997; 6(2): 104–114.

[45] Kwakye TO, Welbeck EE, Owusu GMY. Determinants of intention to engage in sustainability accounting & reporting (SAR): The perspective of professional accountants. Int. J. Corp. Soc. Responsib. 2018; 3: 11–24.

[46] Setthasakko W. Barriers to implementing corporate environmental responsibility in Thailand: a qualitative approach. International Journal of Organizational Analysis. 2017; 17(3): 169–83.

[47] Olaoye CO, Olanipekun CT. Impact of forensic accounting and investigation on corporate governance in Ekiti state. Journal of Accounting, Business and Finance Research. 2018; 4(1): 28–36.

[48] Yakhou M, Dorweiler VP. Environmental accounting: An essential component of business strategy. Business Strategy and the Environment. 2014; 13(2): 65–77.

[49] Molina JF, Claver-Cortés E, López-Gamero MD, Tarí JJ. Green management and financial performance: A literature review. Management Decision. 2009; 47(7): 1080–1100.

[50] Latan H, Jabbour C, De Sousa J. Effects of environmental strategy, environmental uncertainty and top management’s commitment on corporate environmental performance: The role of environmental management accounting. Journal of Cleaner Production. 2018; 180: 297–306.

[51] Zaid AA, Jaaron AA, Bon AT. The impact of green human resource management and green supply chain management practices on sustainable performance: An empirical study. Journal of Cleaner Production. 2018; 204: 965–979.

[52] Dibrell C, Craig JB, Kim J, Johnson AJ. Establishing how natural environmental competency, organizational social consciousness, and innovativeness relate. Journal of Business Ethics. 2015; 127(3): 591–605.

[53] Henri JF Journeault M. Eco-control: The influence of management control systems on environmental and economic performance. Accounting, Organizations and Society. 2021; 35(1): 63–80.

[54] Jermsittiparsert K, Siam M, Issa M. Do consumers expect companies to be socially responsible? The impact of corporate social responsibility on buying behavior. Uncertain Supply Chain Management. 2019; 7(4): 741–752.

[55] Klassen RD, Whybark DC. The impact of EMA on manufacturing performance. Academy of Management Journal. 2019; 42: 599–615.

[56] Johnstone L. Environmental management decisions in CSR‐ based accounting research. Corporate Social Responsibility and Environmental Management. 2018; 25(6): 1212–1222.

[57] Henseler J, Ringle CM, Sarstedt M. A new criterion for assessing discriminant validity in variance-based structural equation modelling. Journal of the Academy of Marketing Science. 2015; 43(1): 115–135.

[58] Le GC, Boyer AL, Poupeau F. Environmental regulations and sustainable mining in the semi-arid American southwest: Perspectives from the national environmental protection act process for the Rosemont mine project (Arizona). Regional Environmental Change. 2019; 19(2): 501–513.

[59] Hair JF, Hult G, Ringle C. Sarstedt M. A Primer on Partial Least Squares Structural Equation Modeling (PLS-SEM). USA: Sage Publications, 2013.

